# Qualified Practitioners in Belgium

**Published:** 1895-09

**Authors:** 


THE qualified practitioners in Belgium number 2,950, 1 to 2,122; midwives number 2,372, 1 to 2,640.

